# Secondary urethral sphincter function of the rabbit pelvic and perineal muscles

**DOI:** 10.3389/fnins.2023.1111884

**Published:** 2023-02-16

**Authors:** Ana G. Hernandez-Reynoso, Farial S. Rahman, Brian Hedden, Francisco Castelán, Margarita Martínez-Gómez, Philippe Zimmern, Mario I. Romero-Ortega

**Affiliations:** ^1^Department of Bioengineering, The University of Texas at Dallas, Richardson, TX, United States; ^2^Department of Biomedical Engineering and Biomedical Sciences, University of Houston, Houston, TX, United States; ^3^Departamento de Biología Celular y Fisiología, Unidad Foránea Tlaxcala, Instituto de Investigaciones Biomédicas, Universidad Nacional Autónoma de México, Tlaxcala, Tlaxcala, Mexico; ^4^Centro Tlaxcala de Biología de la Conducta, Universidad Autónoma de Tlaxcala, Tlaxcala City, Mexico; ^5^Department of Urology, The University of Texas Southwestern Medical Center, Dallas, TX, United States

**Keywords:** targeted neuromodulation, electrical stimulation, bioelectronic medicine, pelvic floor stimulation, urinary incontinence

## Abstract

Perineal and pelvic floor muscles play an important role in continence by providing mechanical support to pelvic organs. It is also known that the pubococcygeus muscle (PcM) contracts in the storage phase and is inactive during voiding, while the bulbospongiosus muscle (BsM) is active during the voiding phase. Recent evidence suggested an additional role of these muscles in supporting urethral closure in rabbits. However, the individual role of perineal and pelvic muscles as urethral sphincters is not well-defined. Here we evaluated the individual, sequential and synergistic roles of the PcM and BsM in assisting urethral closure and defined the optimal electrical stimulation parameters that can effectively contract these muscles and increase the urethral pressure (P_*ura*_) in young nulliparous animals (*n* = 11). Unilateral stimulation of either the BsM or PcM at 40 Hz induced modest increases in average P_*ura*_ (0.23 ± 0.10 and 0.07 ± 0.04 mmHg, respectively). Investigation on the changes in P_*ura*_ evoked by stimulation frequencies between 5 and 60 Hz show that sequential contralateral PcM-BsM activation at 40 Hz induced a 2-fold average P_*ura*_ increase (0.23 ± 0.07 mmHg) compared to that evoked by PcM stimulation. Simultaneous activation of PcM and BsM at 40 Hz also showed an increased average P_*ura*_ (0.26 ± 0.04 mmHg), with a 2-fold increase in average P_*ura*_ observed during the unilateral sequential PcM-BsM stimulation at 40 Hz (0.69 ± 0.2 mmHg). Finally, stimulation at 40 Hz of the bulbospongiosus nerve (BsN) induced an approximate 4-fold increase in average P_*ura*_ (0.87 ± 0.44 mmHg; *p* < 0.04) compared to that elicited by BsM stimulation, confirming that direct nerve stimulation is more effective. Together, this study shows that in the female rabbit, both perineal and pelvic muscles support of the urethral function during continence, and that unilateral stimulation of the BsN at 40–60 Hz is sufficient to achieve maximal secondary sphincter activity. The results also support the potential clinical value of neuromodulation of pelvic and perineal nerves as bioelectronic therapy for stress urinary incontinence.

## 1. Introduction

In the female pelvis, pelvic organs are supported by several tissues including fascia, ligaments and muscles, either superficial (ischiocavernosus, bulbospongiosus) or deep (pubococcygeus, puborectalis, iliococcygeus and coccygeus) (Henry et al., 1982; DeLancey, 2016; Giraudet et al., 2018). The puborectalis and pubococcygeus muscles wrap the rectum and form a U-shaped sling which pulls the vagina and bladder neck toward the pubic bone, reinforcing the striated structures of the urethra and serving as a secondary sphincter in urinary and fecal continence (Manzo et al., 1997; Silva et al., 2016). Electromyography of the pubococcygeus muscle recorded bilaterally in young continent women during micturition shows phasic activity, that changes to a tonic pattern during bladder filling, confirming their role in storage and voiding (Deindl et al., 1994). Congruently, partial injury and denervation of pelvic floor muscles which often occurs after childbirth through direct tissue compression, stretch, and devascularization, cause loss of urethral and bladder support which contributes to stress urinary incontinence (SUI) (DeLancey, 1994; Alperin et al., 2017; Mathew et al., 2019), a condition affecting approximately 35% of women in the United States (Mckellar and Abraham, 2019; U.S. Census, 2022), with no effective pharmaceutical treatment available.

Therapeutical stimulation of the pelvic floor muscles for SUI symptom alleviation have been investigated using transcutaneous, vaginal, or rectal electrical stimulators. However, their efficacy is controversial, with some studies reporting subjective symptom improvement and others reporting no benefits (Ismail et al., 2009; Dmochowski et al., 2019; Peng et al., 2019). Severe cases are recommended for surgical intervention to provide support to the pelvic floor using mid-urethral slings with synthetic sub-urethral tape or mesh (Oliphant et al., 2009; Dwyer and Karmakar, 2019), which remains the most common surgical procedure for patients with SUI with success rate of 72–77% at 24 months (Imamura et al., 2019). Unfortunately, 12% of those implanted with SUI slings suffer from at least one serious adverse event such as pain, mesh exposure, dyspareunia, voiding dysfunction, urge incontinence, vaginal wall erosion, or recurrent urinary tract infections (Gomes et al., 2017; Gurol-Urganci et al., 2018; FDA, 2019; Keslar et al., 2020), and approximately 4% of patients have to adjust or remove the implant 60 months after initial surgery (Clancy et al., 2019; Brennand et al., 2020). Understanding the anatomy and physiology of individual components will pave the way for the development of personalized diagnosis and more effective treatment options in pelvic floor disorders.

Studies have investigated the function of different pelvic floor muscles in micturition in the adult female rabbit model and found that the pubococcygeus muscle (PcM) is active in the storage phase, and inactive during voiding, while the bulbospongiosus muscle (BsM) activity increases during the voiding phase (Corona-Quintanilla et al., 2009, 2014). Our previous study validated that in this animal model, multiparity and aging cause damage to both perineal and pelvic floor nerves indicated by an approximated 51 and 32% reduction in myelination in the bulbospongiosus nerve (BsN) and pubococcygeus nerve (PcN), respectively, which contribute to reduced urethral pressure and bladder efficiency suggesting a direct role in urethral closure in addition to that in voiding (Hernandez-Reynoso et al., 2021). This study also showed that acute neuromodulation of the BsN at 2–20 Hz improved urethral closure pressure and voiding efficiency. However, the individual role of perineal and pelvic muscles in urethral closure and the optimal parameters for electrical modulation of this nerves which contribute to average maximal urethral pressure, are unknown, and are needed to develop more effective neuromodulation therapies for pelvic floor disorders including stress urinary incontinence. Therefore, in this study we compared the function of PcM and BsM stimulations as secondary sphincters and defined the optimal intramuscular stimulation parameters in these perineal and pelvic muscles to effectively increase urethral pressure (P_*ura*_) in young nulliparous rabbits. Finally, we compared the efficacy of direct muscle stimulation against neuromodulation of their respective nerve targets on urethral closure to optimize the therapeutic effects.

## 2. Materials and methods

### 2.1. Ethics statement

All animal experiments were approved by the University of Texas Southwestern Medical Center Institutional Animal Care and Use Committee (Protocol APN 2019-102575-USDA) and in accordance with ARRIVE guidelines.

### 2.2. Animal use

New Zealand white rabbits were used due to their fully developed pelvic floor muscles and well-defined pelvic floor activation pattern (Corona-Quintanilla et al., 2009; López-García et al., 2016) that is suggested to resemble previously reported human functions (DeLancey, 2016). A total of 11 young female rabbits (Oryctolagus cuniculus) (4.91 ± 0.16 months old and 3.47 ± 0.13 kg) were used in this study. Animals were induced with an injection of intramuscular ketamine (35 mg/kg) and xylazine (5 mg/kg) and maintained throughout the procedure *via* inhaled oxygen (2 L/min) mixed with isoflurane (1–3%) delivered *via* a ventilator. While this gas anesthetic has known effects on the micturition reflex, it does not seem to affect direct urethral closure by pelvic floor muscles (Julia-Guilloteau et al., 2007). Analgesia throughout the procedure was achieved with a dose of intramuscular buprenorphine HCL (0.05 mg/kg), as needed. The rabbit was given intravenous normal saline at a rate of 25 mL/min (Normasol-R). Animals were euthanized at the end of the experiment with an overdose of intravenous (120 mg/kg) pentobarbital sodium and phenytoin sodium (Euthasol).

### 2.3. Surgical procedure and instrumentation

**FIGURE 1:**
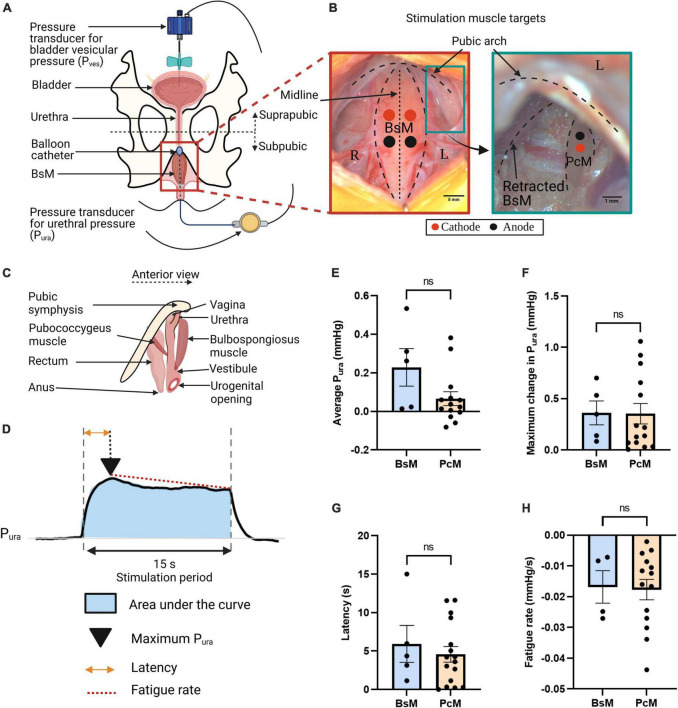
Similar contribution of urethral closure by perineal and pelvic muscles. **(A)** Schematic of cystometry instrumentation; top view. **(B)** Representative pictures of perineal bulbospongiosus muscle (BsM) (left) and pelvic pubococcygeus muscle (PcM) (right), with position of cathode (red dot) and anode (black dot) needle electrodes indicated. **(C)** Schematic of BsM and PcM in rabbit; anterior view. **(D)** Representative urethral pressure P_ura_ trace during simultaneous BsM+PcM stimulation illustrating quantified variables. Comparison of **(E)** normalized area under the curve or average P_ura_, **(F)** maximum P_ura_ increase, **(G)** latency period, and **(H)** fatigue rate, evoked by independent BsM and PcM stimulations at 40–60 Hz (BsM *n* = 5, PcM *n* = 7). Data is reported as mean ± SEM.

The bladder was partially emptied by manually pressing down on it. The bladder was then exposed by making a 4 cm upward incision above the pelvic crest. The fascia beneath the skin was dissected to expose the abdominal muscles. An incision was made at the midline (Linea Alba) to expose the bladder inside the intraperitoneal space. Then, a 2 cm downward incision was made from the pubic arch to expose the pelvic floor muscles. The fascia beneath the skin was dissected from the skin until the BsM was visible (Figure 1B). The connective and adipose tissues underneath the pubic arch were dissected exposing the outer side vaginal canal. Both the BsM and vaginal canal were carefully retracted laterally to not induce muscle damage to expose the PcM.

### 2.4. Vesical pressure transducer implantation

A 21-G butterfly needle was inserted into the bladder apex and secured using a purse string suture (Ethicon Perma-hand 4-0 silk suture) connected to a disposable BP pressure transducer (ADInstruments MLT0699) to record suprapubic vesical bladder pressure (P_*ves*_) as denoted in Figure 1A. The abdominal muscle, fascia and skin were sutured back together to acquire true P_*ves*_ recording.

### 2.5. Intramuscular electrode implantation for muscle stimulation

For direct muscle stimulation (*n* = 7), a total of eight perfluoroalkoxy-coated tungsten wires [A-M Systems, Inc. of diameter: 0.002 inch (bare) and 0.0040 inches (coated); Catalog # 795500] acting as stimulating electrodes were stripped at the tip. 30-gauge needles were used for insertion of the wired electrodes at the belly of the right and left sides of the PcM and BsM, and removed afterward leaving the electrodes in the muscle. Two wires per right and left muscles were implanted: cathode and anode electrodes as denoted by the red and black dots in Figure 1B for targeted bilateral stimulation.

### 2.6. Extraneural electrode implantation for nerve stimulation

For direct nerve stimulation, the BsN and PcN were identified by their direct anatomical innervation to the BsM and PcM, respectively. The BsN, for example, was found lateral to the clitoralis nerve and the terminal nerve endings were observed innervating the BsM. Both nerves, BsN and PcN, were surgically exposed using blunt microdissection scissors and glass rods. Hooked tungsten wire electrodes were implanted in the BsN (*n* = 2) and the PcN (*n* = 2) and secured in place using a sealant (Kwik-Cast Silicone Sealant, World Precision Instruments). In addition, a wired neural stimulator (wired NeuroClip, RBI) was implanted directly in the BsN (*n* = 1).

### 2.7. Urethral pressure transducer placement

A balloon catheter (MILA Anal Sac Balloon Catheter 4fr × 17.5 cm) was advanced 3 cm through the cloaca to the urethra and connected to a second pressure transducer (ADInstruments MLT844 with a MEMSCAP 844-28 disposable dome coupler) to measure P_*ura*_. Both pressure transducers were calibrated before insertion using a pressure gauge between 0 and 20 mmHg and recorded (1 kHz) simultaneously during stimulation using an acquisition system (ADInstruments PowerLab 4/26 and two ADInstruments bridge amplifiers) *via* the ADInstruments LabChart™ Software.

### 2.8. Electrical stimulation

Target muscles were stimulated with cathodic, monophasic, 20 μs pulses using an A-M Systems Model 2100 isolated pulse stimulator. The stimulating threshold current for the muscle targets was tested for each animal (approximately 0.5–0.8 mA) and the stimulating current was set at 2 mA above this threshold (Stimulating current = threshold current + 2 mA). This was sufficient to cause maximal recruitment of muscles at the given parameters. Stimulations were applied for 15 s at frequencies 5, 10, 20, 40, to 60 Hz. Different muscle activation patterns were evaluated: (1) BsM, (2) PcM, (3) BsM→PcM (sequential), and (4) BsM+PcM (simultaneous). The sequential pattern is expected to mimic the physiological patterns of PFM activation during storage followed by voiding. For the sequential and simultaneous stimulation, three additional configurations were explored at a fixed frequency of 40 Hz: unilateral (either right or left), bilateral, and contralateral (stimulation of the PcM on one side, and BsM on the other), designed to reduce fatigue. The 40 Hz stimulation frequency was selected based on initial results showing efficient muscle response and urethral closure at this frequency. Separate studies were performed with direct nerve stimulation of the BsN and PcN at the same frequency range and at threshold currents at which corresponding muscle response was observed (0.4–1 mA) to compare the effect of muscle vs. nerve stimulation. Two minutes of rest were given between each stimulation, and between each pattern, and 15 min rest were given between sessions.

### 2.9. Data analysis and quantification

Pressure data were recorded with a 15 s baseline window followed by 15 s stimulations for each target or parameter tested. Data was saved in ADInstruments proprietary format, exported to text files (.txt), processed in MATLAB R2020b (version 9.9.0.1495850, license 706581) and detrended by removing the baseline mean from the signal and applied a finite impulse response low pass filter with a 1 Hz stopband (60 dB attenuation and 0.99 steepness). The area under the curve was calculated using MATLAB’s trapezoidal numerical integration function (trapz). The stimulation was triggered manually which resulted in some milliseconds variability, thus this data was normalized by dividing it over effective stimulation time, resulting in an integrated average (mmHg) of the P_*ura*_ referred to as average P_*ura*_. The maximum P_*ura*_ value was identified during the stimulation period and the time between the stimulation onset and this peak value was defined as the latency period. Finally, fatigue rate was defined as the slope of the linear polynomial regression between the time of the maximum P_*ura*_ and the end of the stimulation window. For sequential stimulation pattern, the time from stimulation start to end for both muscles (30 s approximately) were considered as the entire stimulation period for quantification purposes, with the delay between the stimulation period (3–5 s) included in average P_*ura*_ and latency calculations. The overall maximum value obtained during the sequential stimulation of both targets was identified as the maximum P_*ura*_ value and used for latency and fatigue calculations. Trials with negative maximum P_*ura*_ were discarded as non-responsive trials. Table 1 summarizes the variables quantified from the pressure data, formulas, and interpretation.

**TABLE 1:** Summary of quantified variables used to determine electrical stimulation efficacy.

Variable quantified	Formula	Meaning
Maximum increase in P_ura_ during stimulation (mmHg)	*maximumP*_*ura*_*duringstimulation*−*meanP*_*ura*_*atbaseline*	Maximum increase in urethral closure pressure during stimulation
Normalized area under the curve (mmHg)	$\frac{\int_{t_{stimstart}}^{t_{stimend}}y=f\left(x\right)}{t_{stimend}-t_{stimstart}}$	Average urethral closure pressure during stimulation
Time to maximum P_ura_ (s)	*timeatmaximumP*_*ura*_−*starttime*	Latency period
Slope between maximum P_ura_ and P_ura_ at stimulation end (mmHg/s)	$\frac{P_{ura}atstimulationend-maximumP_{ura}}{t_{stimend}-t_{\max Pura}}$	Fatigue rate

### 2.10. Statistical analysis

**FIGURE 2:**
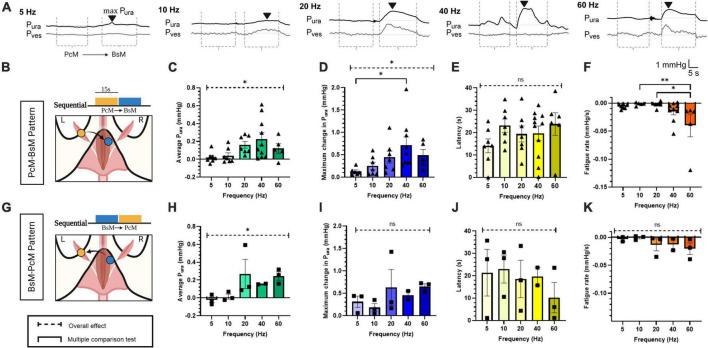
Effects of pelvic-perineal order of sequential recruitment in urethral pressure. **(A)** Representative P_ura_ (black trace) and P_ves_ (gray trace) recordings during baseline and stimulation at frequencies ranging 5–60 Hz for PcM→BsM stimulation. The stimulation windows for each individual muscle are indicated by dotted lines (----). The black arrowhead (▼) labels the max P_ura_ and indicates latency from onset. **(B,G)** Schematic of stimulation patterns. Comparison of **(C,H)** average P_ura_, **(D,I)** maximum P_ura_ increase, **(E,J)** latency period estimated from the bulbospongiosus muscle (BsM) evoked response, and **(F,H)** fatigue rate is shown for PcM→BsM [*n* = 7; **(B–E)**] and BsMPcM [*n* = 3; **(F–I)**] stimulation sequence. with results reported as mean ± SEM. Statistical analysis conducted using Brown-Forsythe and Welch one-way ANOVA tests with Dunnett’s T3 multiple comparison tests for normal data sets **(C,E,H,J,K)**, and with Kruskal-Wallis with Dunn’s multiple comparison for non-Gaussian distributions **(D,F,I)** (α = 0.05, *p < 0.05, **p < 0.01).

**FIGURE 3:**
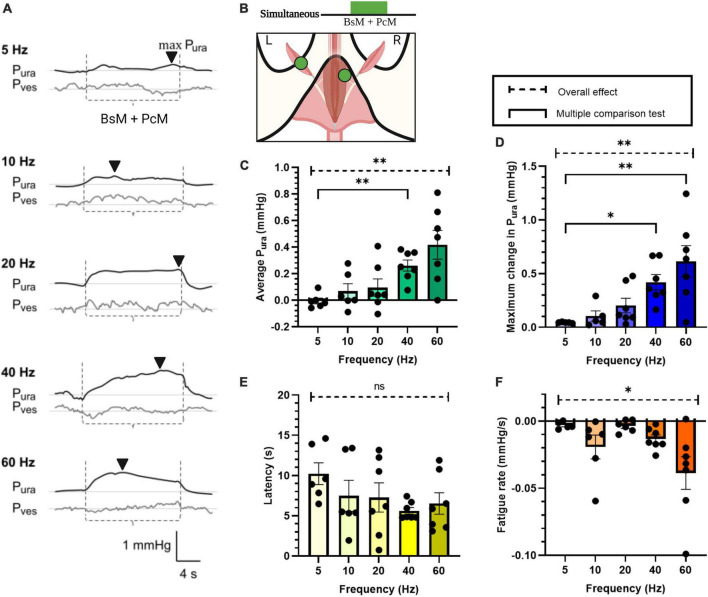
Increase in urethral closure pressure by simultaneous BsM+PcM stimulation. **(A)** Representative P_ura_ (black trace) and P_ves_ (gray trace) recordings during baseline and stimulation at frequencies ranging 5–60 Hz. The stimulation windows for each individual muscles are denoted between the dotted lines (----) and the max P_ura_ is indicated using the black arrowhead (▼). **(B)** Stimulation schematic. Quantification of **(C)** average P_ura_, **(D)** maximum P_ura_ increase, **(E)** latency period, and **(F)** fatigue rate, with *n* = 7 sample size and results reported as mean ± SEM. Statistical analysis conducted using Brown-Forsythe and Welch one-way ANOVA tests with Dunnett’s T3 multiple comparison tests for normal data sets **(C,F)**, and with Kruskal-Wallis with Dunn’s multiple comparison for non-Gaussian distributions **(D,E)** (α = 0.05, *p < 0.05, **p < 0.01).

**FIGURE 4:**
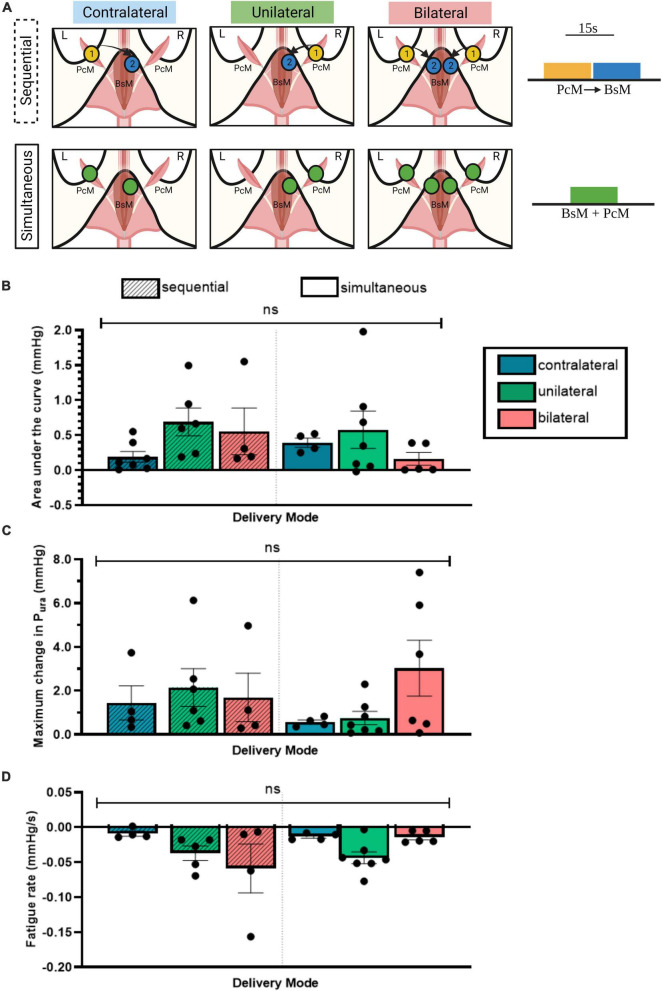
Unilateral PcM→BsM stimulation is sufficient in evoking maximal urethral closure. **(A)** Schematic of the six stimulation configurations investigated using two stimulation patterns, sequential and simultaneous. Quantification and comparison of effect of each stimulation pattern on: **(B)** average P_ura_, **(C)** maximum change in P_ura_, and **(D)** fatigue rate. Results reported as mean ± SEM (*n* = 5). No significant difference was observed between the different configurations (ordinary two-way ANOVA, Tukey’s multiple comparison, alpha = 0.05).

**FIGURE 5:**
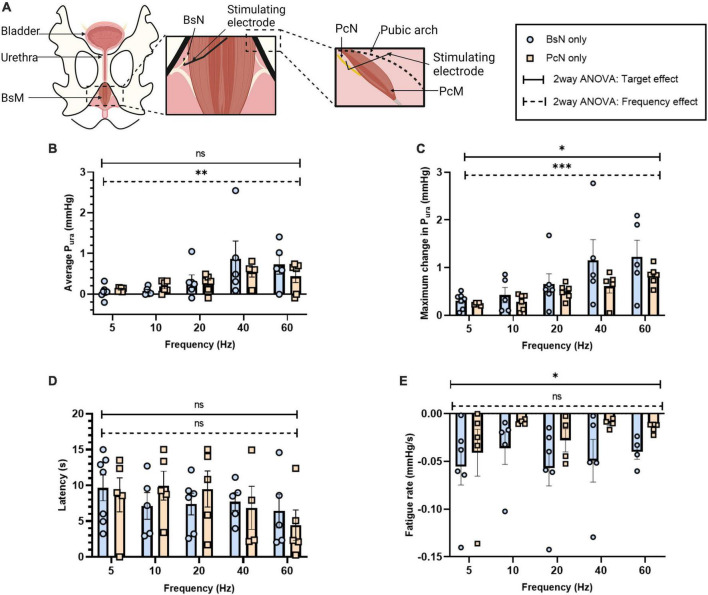
Effect of direct electrical stimulation of bulbospongiosus nerve (BsN) and pubococcygeus nerve (PcN). **(A)** Experimental set-up illustrating BsN and PcN surgical exposure, and electrode placement. Quantification of **(B)** average P_ura_, **(C)** maximum P_ura_ increase, **(D)** latency period, and **(E)** fatigue rate. Results reported as mean ± SEM, with sample size for BsN *n* = 3 and PcN *n* = 2, with repetitive measurements from single animals. Statistical analysis conducted with ordinary two-way ANOVA, showing both target and frequency effect on significance testing α = 0.05, *p < 0.05, ^**^p < 0.01, ^***^p < 0.005).

Sample sizes were calculated using the G*Power software (Faul et al., 2007) to achieve an 80% power. Area under the curve of preliminary data (*n* = 3) was used to determine that the effect size = 0.54, resulting in an *n* = 7 needed per frequency. Statistical analysis was performed in RStudio version 1.3.1093 (R version 4.0.3) and GraphPad Prism version 9.4.1 for Windows (GraphPad Software, San Diego, CA, USA). The ROUT method (Q = 2%) was used to exclude outliers (see Supplementary Table 1). Their distribution was calculated using the Shapiro-Wilk normality test (α = 0.05). To compare results between BsM and PcM (Figure 1), unpaired two-tailed Mann-Whitney test was performed for non-normal distributions and unpaired *t*-test with Welch’s correction was performed for normal data sets. To determine the effect of frequency on each parameter (Figures 2, 3), data sets with normal distributions were tested using Brown-Forsythe test ANOVA with Dunnett’s multiple comparison, while non-normal data were tested using Kruskal Wallis with Dunn’s multiple comparison. Two-way ANOVA with Tukey *post-hoc* test for multiple comparisons was conducted for data sets having two independent variables (Figures 3–5). Results are reported as mean ± SEM. Statistical significance: *p < 0.05, ^**^p < 0.01, ^***^p < 0.005.

## 3. Results

### 3.1. Individual perineal and pelvic muscles contribute to mild increase in urethral pressure

Old multiparous rabbits with SUI-like deficits in urethral pressure and bladder efficiency showed damage in both bulbospongiosus and pubococcygeus nerves (BsN and PcN, respectively), and BsN acute stimulation in these animals increased the urethral pressure (P_*ura)*_ and improved bladder efficiency (Hernandez-Reynoso et al., 2021), raising the possibility that both pelvic and perineal muscles might contribute to urethral closure in this animal model. This hypothesis is investigated by directly stimulating these targets and measuring the effect on P_*ura*_. A suprapubic pressure-sensor catheter was used to measure vesical bladder pressure (P_*ves*_) and a subpubic one for urethral pressure (Figure 1A). The muscles targets were exposed and directly stimulated using wire electrodes at 40–60 Hz (*n* = 7) (Figure 1B). The anatomical location of these peripheral and pelvic floor muscles in relation to the urethral sphincter (Figure 1C), suggested that electrical activation of each target could move or close the urogenital hiatus. The effect of muscle stimulation was evaluated from: (a) the P_*ura*_ normalized area under the curve indicating average urethral closure pressure, (b) the maximum increase in P_*ura*_, (c) the latency time for maximum urethral closure, and (d) muscle fatigue rate (Figure 1D). Unilateral stimulation of either the BsM or the PcM induced modest increases in average P_*ura*_ closure (BsM: 0.23 ± 0.1 mmHg, PcM: 0.07 ± 0.04 mmHg) that were not statistically different. The evoked maximum P_*ura*_ was comparable for each muscle (BsM: 0.36 ± 0.12 mmHg, PcM: 0.35 ± 0.10 mmHg), and no differences were observed either in latency (BsM: 5.92 ± 2.40 s, PcM: 4.57 ± 1.02 s), or in fatigue rate (BsM: –0.02 ± 0.01 mmHg/s, PcM: –0.02 ± 0.003 mmHg/s; Figure 1H). This data supports a similar role for urethral closure for these two pelvic and perineal muscles.

### 3.2. Sequential activation of BsM and PcM does not have a synergistic effect on P_*ura*_

The physiological activation of the BsM and PcM is asynchronous during the rabbit micturition cycle; the PcM is active during the storage phase, while the BsM activity increases during voiding (Corona-Quintanilla et al., 2009). Thus, we evaluated if sequential activation of these muscles impacts their function in assisting urethral closure. The possibility of muscle co-activation by artifact volume conduction was minimized by the contralateral (_c_) stimulation of each target. In this study, the maximum P_*ura*_ was observed primarily during BsM stimulation, with BsM stimulation resulting in a significantly greater probability (65 ± 11%) of evoking a maximum P_*ura*_ increase (*p* < 0.04; Figure 2A). We also noted an effect of stimulation frequencies above 20 Hz in increasing average P_*ura*_ (*p* < 0.05; Figure 2C *n* = 7), and of maximum P_*ura*_ in the PcM→_c_BsM sequential activation (*p* < 0.05) which was significantly increased (*p* < 0.01) at 40 Hz (0.71 ± 0.18 mmHg) compared to that at 5 Hz (0.12 mmHg; Figure 2D, *n* = 7). Muscle fatigue was observed at higher frequencies (40–60 Hz) after PcM→_c_BsM stimulation (*p* < 0.005; Figure 2F, *n* = 7), but was not significant when the pattern was reversed (BsM→_c_PcM) (Figure 2K, *n* = 3), although fatigue increase was still observed at 60 Hz (–0.04 ± 0.02 mmHg/s) compared to that at 10 and 20 Hz (–0.001 to 0.002 mmHg/s). Evaluation of latencies in evoked responses showed no differences between the two activation pattern sequences (Figures 2E, J). Together, these results indicated that stimulation PcM→_c_BsM or BsM→_c_PcM induced similar urethral closure effects and maximal sphincter-like function when activated at frequencies higher than 20 Hz, without causing significant muscle fatigue after 15 s stimulation in the BsM→_c_PcM stimulation pattern. Stimulation of the BsM produced larger increments in P_*ura*_ compared to that evoked by PcM in both sequences, indicating that the perineal muscle plays a more relevant role as secondary sphincter in the rabbit. This result also showed that the physiological asymmetric and sequential pattern of pelvic and perineal muscle does not seem to alter their functions as secondary sphincters.

### 3.3. Pelvic-perineal muscle co-activation increase their urethral pressure effect

The effect of PcM+BsM simultaneous contraction on P_*ura*_ was evaluated at different frequencies (Figure 3, *n* = 7). At lower frequencies (5–20 Hz), we observed a mild and transient effect (0.01–0.1 mmHg) while frequencies above 40 Hz consistently evoked a significant average increase in P_*ura*_ (0.26 ± 0.04 mmHg at 40 Hz, *p* < 0.003). This result indicated that simultaneous stimulation of pelvic and perineal muscles doubles their secondary sphincter effect on the urethra compared to individual muscle or sequential stimulation, particularly at 40–60 Hz (Figure 3C). The maximum P_*ura*_ also showed significant increase (*p* < 0.002) at 40–60 Hz (0.42–0.61 mmHg) when compared to 5 Hz (0.05 ± 0.001 mmHg) and 20 Hz (0.20 ± 0.07 mmHg; *p* < 0.03 and *p* < 0.005, respectively). Evaluation of P_*ura*_ response latencies did not show significant changes with increasing frequencies although a 50% reduction at 40 Hz (5.61 ± 0.41 s) compared to that at 5 Hz (10.22 ± 1.35; *p* < 0.04) was observed. As expected, a significant effect on fatigue rate was observed at higher stimulation frequencies (*p* < 0.05), with a 10-fold increase in fatigue rate at 60 Hz (–0.04 ± 0.01 mmHg/s) compared to 5 Hz (–0.003 ± 0.001 mmHg/s) and 20 Hz (–0.004 ± 0.01 mmHg/s).

### 3.4. Unilateral pelvic-perineal muscle stimulation is sufficient for moderate urethral closure

To evaluate if there is an effect on laterality of stimulation, we stimulated the BsM at 40 Hz unilaterally, contralaterally, or bilaterally, and either with sequential (PcM→_*c*_BsM) or simultaneous stimulation (PcM+BsM) patterns (Figure 4A) to compare the evoked increment in urethral pressure (*n* = 5). Unilateral sequential stimulation of the PcM→BsM resulted in a 0.69 ± 0.2 mmHg average P_*ura*_ value, which was comparable to that obtained after bilateral sequential stimulation (0.56 ± 0.26 mmHg) seem more effective compared to contralateral sequential activation of these muscles (0.19 ± 0.08 mmHg; Figure 4B), although these values are not statistically significant. Simultaneous muscle recruitment produced similar average P_*ura*_ values unilaterally (0.58 ± 0.27 mmHg) but they decreased after bilateral (0.16 ± 0.09 mmHg) or contralateral stimulation (0.39 ± 0.06 mmHg). Evaluation of the maximum P_*ura*_ yielded similar results with maximal contraction observed after unilateral sequential stimulation (2.15 ± 0.87 mmHg), which was comparable to that observed after bilateral simultaneous recruitment (3.03 ± 1.27 mmHg; Figure 4C). Moderate fatigue rates were noted in the unilateral PcM→BsM stimulation mode (–0.05 ± 0.03 mmHg/s) compared to that in contralateral sequential stimulation, and they increased after bilateral sequential stimulation (–0.01 ± 0.003 mmHg/s; Figure 4D). These results indicate that unilateral BsM stimulation induced an effective urethral pressure increment with minimal muscle fatigue after 15 s of continuous stimulation.

### 3.5. Direct perineal nerve stimulation induced efficient urethral pressure

**FIGURE 6:**
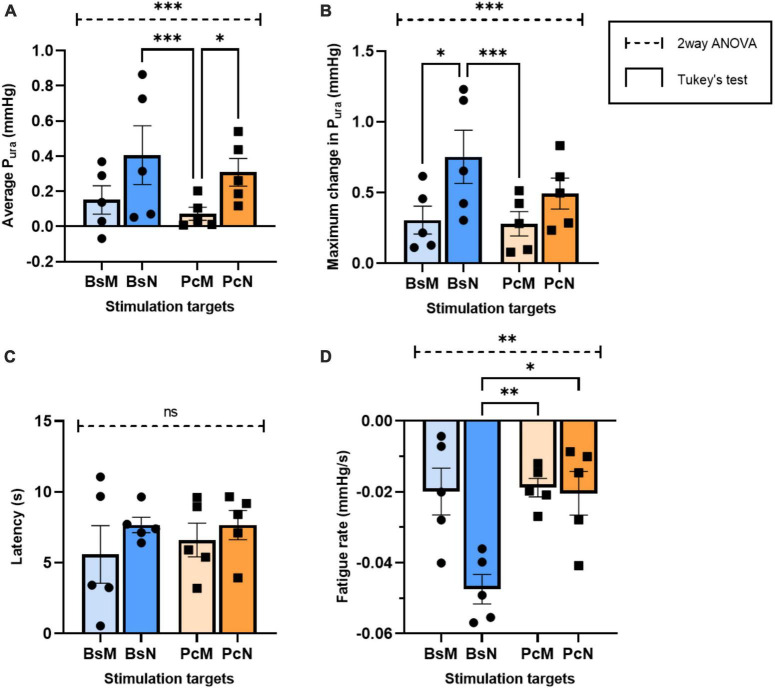
Comparative analysis of urethral closure for direct electrical stimulation between nerve and associated muscles. Quantification of means of **(A)** average P_ura_, **(B)** maximum P_ura_ increase, **(C)** latency period, and **(D)** fatigue rate, with error bars representing the SEM. Statistical analysis conducted with ordinary two-way ANOVA, followed by Tukey’s multiple comparison tests (α = 0.05, *p < 0.05, ^**^p < 0.01, ^***^p < 0.005).

It is expected that direct perineal nerve stimulation will contract the innervated muscle more effectively, but the extent of which it will increase the P_*ura*_ over that evoked by intramuscular stimulation has not been reported. We measured the changes in urethral pressure evoked by the stimulation of individual nerve targets BsN (*n* = 3) and PcN (*n* = 2) at 5–60 Hz frequencies (Figure 5). Direct neuromodulation of BsN showed an approximate 3-fold increase in average P_*ura*_ at 40 Hz (0.87 ± 0.44 mmHg) and 60 Hz (a 0.73 ± 0.24 mmHg; *p* < 0.04), compared to that observed after BsM stimulation (see Figure 1E). Maximum P_*ura*_ induced by BsN stimulation reached 1.52 ± 0.43 and 1.23 ± 0.35 mmHg at 40 and 60 Hz, respectively, a 4-fold increment over that observed with direct muscle stimulation (Figure 5B). The latency to maximum P_*ura*_ in response to BsN 40 Hz (7.7 ± 1.24 s) was comparable to that observed after BsM stimulation (Figure 5D). Congruently, a 2-fold increase in mean muscle fatigue rate was observed at 40 and 60 Hz (Figure 5E) after direct nerve stimulation (–0.04 mmHg/s; *p* = 0.02). Stimulation of the PcN also elicited a 5-fold increase in average P_*ura*_ at 40 Hz (0.54 ± 0.12 mmHg) and 60 Hz (a 0.44 ± 0.16 mmHg) compared to that of the PcM (*p* = 0.01; Figure 5B). However, it was only 62% of the pressure observed with direct BsN stimulation, indicating that in the rabbit the BsN plays a slightly more prominent role in urethral closure. This was confirmed by the lower values observed in maximum P_*ura*_ after PcN stimulation, which reached 0.63–0.82 mmHg at 40–60 Hz, an estimated 41.5% of what was observed with direct BsN stimulation (*p* = 0.01; Figure 5C). No differences were observed in latency response in P_*ura*_ after PcN, and stimulation of the former did not significantly change the average muscle fatigue rate (0.01 mmHg/s) at 40–60 Hz (Figure 5E). A direct comparison between the pelvic/perineal muscle and their associated nerve stimulation is shown in Figure 6.

## 4. Discussion

The role of the pelvic muscles as secondary urinary sphincters has been recognized (Yucel and Baskin, 2004), and particularly the role of the levator ani pubococcygeus, has been documented in in animal models of SUI (Manzo et al., 1997; Martínez-Gómez et al., 2011), and in women suffering from this condition (Smith et al., 1989; DeLancey, 1994; Heesakkers and Gerretsen, 2004; Giraudet et al., 2018; Falah-Hassani et al., 2021). However, the role of perineal muscles such as the BsM are associated with sexual function. Their potential role as secondary urethral was suggested by our recent studies in nerve injury and neuromodulation in mature multiparous rabbits (Hernandez-Reynoso et al., 2021). In women, the BsM is attached to the clitoral hood, but it is also interconnected with the superficial transverse perineal muscle, and the external anal sphincter (EAS) (Baramee et al., 2020), and it is located close to the external urinary sphincter in men (EUS) (Kinugasa et al., 2013). In female rabbits, the BsM runs along the perineal vagina, just caudal to the urethral opening of the rabbit’s cloaca (Cruz et al., 2002). This anatomical configuration support to the notion that this muscle might also play a role in urinary continence (Rehder et al., 2016).

Previous studies of urinary and bladder functions in animal models have shown that the BsM is active during expulsion of seminal fluids in male rats (Tanahashi et al., 2012) and that urethral dysfunction correlates with abnormalities in both BsM and PcM (Fajardo et al., 2008; López-García et al., 2014, 2016; Corona-Quintanilla et al., 2020). Our study used acute electrical stimulation of the BsM and the PcM to evaluate the functional roles of these muscles as secondary sphincters in the female rabbit. We compared single muscle stimulation, simultaneous co-activation, sequential (PcM→BsM), and reversed sequential (BsM→PcM) stimulation patterns over a range of 5–60 Hz to evaluate their effect on urethral closure. Single unilateral PcM or BsM activation evoked similar levels of maximal P_ura_, indicating that in the rabbit these pelvic and perineal muscles contribute or assist as secondary sphincters. Sequential activation following the physiological pattern during micturition (PcM→BsM) and simultaneous contraction of these muscles produced an increase in average P_ura_, consistent with the notion of independent and equal ability of these muscles to support the urethral function in this animal model. Moreover, evaluation of the unilateral, contralateral, or bilateral muscle activation revealed the unexpected finding that unilateral stimulation of the pelvic-perineal muscle sequence (PcM→BsM) doubled the pressor force compared to that of bilateral simultaneous activation, revealing that a single side stimulation is sufficient for maximal activation of their secondary sphincter function.

Studies in peripheral nerve injury repair by Ju and colleagues found that volume conduction evoked by non-invasive methods do not match the faster recovery and functional outcomes observed after direct nerve stimulation (Ju et al., 2020). This suggested that direct stimulation of the PcN and/or BsN could produce higher P_ura_ by optimally contracting the perineal and pelvic muscles. Our results confirmed that stimulation of the BsN or PcN showed a 3–5-fold increase in average P_ura_ value at 40 Hz compared to BsM or PcM stimulation, respectively. However, stimulation of the BsN was 40% better in evoking a maximal P_ura_ response, compared to the PcN, suggesting that in the rabbit the BsN plays a slightly more prominent role in urethral closure.

Despite the 10 min resting period between stimulations, activation of these muscles, either directly or indirectly, resulted in some degree of muscle fatigue. This study showed that contralateral simultaneous stimulation of pelvic and perineal muscles can avoid to some extent the mild muscle fatigue observed after 15 s stimulation at frequencies ≥40 Hz. Since this study only relied on urethral pressure measurements, further studies will be necessary to determine if a shorter stimulation train or multiple trains with a larger inter-pulse delay can be used to minimize the risk of muscle fatigue.

Frequency analysis for parameters were conducted in the 5–60 Hz range for all stimulation patterns, and consistently found a significant increase in P_ura_ at frequencies above 20 Hz, and optimal at 40–60 Hz. This observation was consistent regardless of the stimulation of the pelvic or perineal targets, and of muscle or nerve stimulated tissues. Current therapeutic solutions for SUI include Kegel exercises or pelvic floor muscle therapy (PFMT) with an efficacy ranging between 35 and 80%, but limited by degree of pelvic floor muscle damage, patient compliance, access to physical therapists and long-term commitment (Rovner and Wein, 2004; McIntosh et al., 2015; Lavelle and Zyczynski, 2016; Dumoulin et al., 2017). Currently available techniques to strengthen the pelvic floor muscles in women with SUI include external electrical stimulation, intravaginal probes and extracorporeal electromagnetic chairs, and while these are non-selective, they have a reportedly 34–70% cure and improvement rates (Dmochowski et al., 2019; Peng et al., 2019; Samuels et al., 2019). Our study suggests that the use of implantable miniature nerve stimulation devices, although more invasive, might offer a direct and effective alternative to activate primary and secondary muscular sphincters, which could be beneficial for the treatment of some types of incontinence. We have previously reported that acute electrical stimulation of the BsN in aging and multiparous rabbits was able to reverse the acute dysfunction in bladder efficiency, presumably caused by partial damage to the pelvic and perineal nerves, and reported a significant benefit of stimulating the BsN at 10–20 Hz compared to 2–5 Hz (Hernandez-Reynoso et al., 2021). The present results indicate that higher stimulation frequencies (40–60 Hz) are needed to achieve tetanic muscle contraction to optimize the evoked urethral closure effect. Furthermore, this study shows that direct unilateral stimulation of the BsN is sufficient and more effective in evoking maximal urethral pressure, compared to simultaneous and bilateral muscle activation. This finding is relevant to the several muscle stimulators for PFMT that have been suggested for the treatment of SUI (Huang et al., 2017; Heesakkers et al., 2018) since due to their cutaneous placement and relatively large sizes (volume between 90 and 150 mm^3^), are not efficient or specific, which contribute to the variability of the treatment and compromises its effectiveness. We and others have reported on miniaturized electronics resulting in sub-millimeter stimulators (0.45 mm^3^) (Freeman et al., 2017), which will enable the next generation of minimally invasive procedures for neuromodulation of pelvic floor disorders.

### 4.1. Limitations

The generated urethral pressures by pelvic and perineal muscle activity in this study are relatively small compared to those previously reported for the PcM in rabbits (P_ura_:13 ± 6 mmHg) (Rajasekaran et al., 2012). Whether these changes in urethra pressure are meaningful for continence, and how do they relate that contribute by the internal and external urinary sphincters remains to be determined. It also important to recognize that while optimization of the frequency was evaluated other parameters such as the optimal pulse duration, train duration, stimulating current, and duty cycles remains to be defined. The differences in perineal and pelvic muscle anatomy and function between rabbits and humans deserves consideration. The BsM in the rabbit lays primarily on the dorsal aspect of the vagina and the urethra ends anteriorly, which differs from that in humans in which the BsM is located lateral to the perineal vagina-urethra. In turn, the rabbit PcM contributes in part to movement of the tail but forms a sling structure over the human urethra suggesting a more direct role on urethral closure compared to the BsM in women. Therefore, further studies are needed in large animal models such as the sheep that better resemble the human anatomy (Urbankova et al., 2017) to evaluate the contribution of these muscles in urethral pressure and the possibly application of neuromodulating these targets in SUI. In addition, variability of the pelvic and perineal innervation of the peri-urethral musculature in most animal models including the rabbit are poorly described. In humans the levator ani which innervates the pelvic floor muscles has five different branching patterns (Rousset et al., 2012; Loukas et al., 2016; Plochocki et al., 2016). Also, the P_ura_ values in this study represent a single point measurement, while in women these are estimated with a profilometry along the urethral canal (Stafford et al., 2020). Thus, a direct correlation to the human physiology while tempting, cannot be directly estimated from this study. further research in larger animal models is needed to better understand the differential effect of perineal and pelvic nerves and muscles as secondary urethral sphincters and as a potential therapy for SUI. Finally, it is known that aging and multiparity result in partial perineal and pelvic nerve injuries (Catanzarite et al., 2018; Burnett et al., 2019) but further understanding the effect of nerve injury of specific pelvic and perineal nerves and muscles, as they relate to urethral closure, are needed.

In summary, the data supports a role for perineal and pelvic muscles in urinary continence, revealed that unilateral stimulation might be sufficient for increasing urethral pressure, and that direct nerve stimulation is more effective compared to muscle recruitment in achieving maximal urethral closure. These findings support the notion that miniaturized bioelectronic therapy for the pelvic and/or perineal muscles might be a potential treatment for stress urinary incontinence.

## Data availability statement

The original contributions presented in this study are included in this article/Supplementary material, further inquiries can be directed to the corresponding author.

## Ethics statement

The animal study was reviewed and approved by University of Texas Southwestern Medical Center Institutional Animal Care and Use Committee (Protocol APN 2019-102575-USDA).

## Author contributions

AH-R: surgeries, design of experiments, data acquisition, quantification and analysis, data interpretation, figures preparation, draft, and revise the manuscript. FR: surgeries, data acquisition, analysis and interpretation, figures preparation, draft, and revise the manuscript. BH: surgeries, data acquisition, and validation. FC and MM-G: conception of research and revise the manuscript. PZ: conception of research, interpretation of data, and revise the manuscript. MR-O: conception of research, design of experiments, surgeries, data acquisition and analysis, draft, and revise the manuscript. All authors contributed to the article and approved the submitted version.

## Supplementary material

The attached file “Supplementary Table 1. Sample size and outliers” provides the table for outlier summaries for each test.
