# Supplementary material for: Secondary urethral sphincter function of the rabbit pelvic and perineal muscles
Source: Front Neurosci. 2023 Feb 16;17:1111884. doi: 10.3389/fnins.2023.1111884 (PMC9978527; doi:10.3389/fnins.2023.1111884)
Supplement: Supplementary file 1 — The attached file “Supplementary Table 1. Sample size and outliers” provides the table for outlier summaries for each test. [file Data_Sheet_1.PDF]

Individual Muscle stimulation

Area under the curve	BsM	PcM
# Y values analyzed	5	16
Outliers	0	2

Max Pura Incrase	BsM	PcM
# Y values analyzed	5	15
Outliers	0	1

Sequential PcM-BsM stimulation

Area under the curve					
Frequency	5 Hz	10 Hz	20 Hz	40 Hz	60 Hz
# Y values analyzed	7	7	7	11	6
Outliers	0	0	0	1	1
Max Pura Incrase					
# Y values analyzed	7	7	7	11	6
Outliers	1	0	0	2	1
Fatigue					
# Y values analyzed	7	7	7	11	5
Outliers	0	2	1	2	0

Sequential BsM-PcM stimulation : no outliers

Simultaneous BsM+PcM stimulation

Area under the curve	5 Hz	10 Hz	20 Hz	40 Hz	60 Hz
# Y values analyzed	7	7	9	13	9
Outliers	0	0	0	1	0
Max Pura Incrase					
# Y values analyzed	7	7	9	13	9
Outliers	1	1	0	1	0
Latency					
# Y values analyzed	7	7	9	13	9
Outliers	0	0	0	1	0
Fatigue					
# Y values analyzed	6	7	9	9	8
Outliers	1	0	2	1	0

Delivery mode analysis

Area under the curve	Unil- Sim	**Unil-Seq**	**Bil- Sim**	**Bil Seq**	**Cont- Sim**	**Con- Seq**
# Y values analyzed	8	6	6	4	5	8
Outliers	1	0	1	0	1	1

Max Pura Incrase	Unil- Sim	**Unil-Seq**	**Bil- Sim**	**Bil Seq**	**Cont- Sim**	**Con- Seq**
# Y values analyzed	8	6	6	4	5	5

Outliers	1	0	0	0	1	1

Fatigue	Unil- Sim	**Unil-Seq**	**Bil- Sim**	**Bil Seq**	**Cont- Sim**	**Con- Seq**
# Y values analyzed	7	6	6	4	5	5
Outliers	0	1	1	0	1	1
